# Improved Pixel-Level Pavement-Defect Segmentation Using a Deep Autoencoder

**DOI:** 10.3390/s20092557

**Published:** 2020-04-30

**Authors:** Rytis Augustauskas, Arūnas Lipnickas

**Affiliations:** Department of Automation, Kaunas University of Technology, 51367 Kaunas, Lithuania; arunas.lipnickas@ktu.lt

**Keywords:** CNN (Convolutional neural networks), deep learning, pavement defects, residual connection, attention gate, atrous spatial pyramid pooling

## Abstract

Convolutional neural networks perform impressively in complicated computer-vision image-segmentation tasks. Vision-based systems surpass humans in speed and accuracy in quality inspection tasks. Moreover, the maintenance of big infrastructures, such as roads, bridges, or buildings, is tedious and time-demanding work. In this research, we addressed pavement-quality evaluation by pixelwise defect segmentation using a U-Net deep autoencoder. Additionally, to the original neural network architecture, we utilized residual connections, atrous spatial pyramid pooling with parallel and “Waterfall” connections, and attention gates to perform better defect extraction. The proposed neural network configurations showed a segmentation performance improvement over U-Net with no significant computational overhead. Statistical and visual performance evaluation was taken into consideration for the model comparison. Experiments were conducted on CrackForest, Crack500, GAPs384, and mixed datasets.

## 1. Introduction

Continuous pavement monitoring can be an extremely tedious task for humans but easy for automated computer-vision (CV)-based systems. As stated in [1], transportation infrastructure is the backbone of a nation’s economy that should be systematically improved. Despite the advantages of modern CV-based systems, real-life applications still meet plenty of challenges. These are usually cases when human experts can identify a surface’s three-dimensional defect from first glance, but classical image analysis techniques still fall behind. Therefore, researchers are constantly seeking new approaches to address these challenges.

In ImageNet competitions [2], it was proven that deep neural network-based solutions surpass classical CV methods in object detection by engaging many layers of data abstraction. A data-driven deep-learning (DL) approach might take into consideration a wider spectrum of cases appearing in a complicated problem than can be constrained by only fundamental knowledge.

Difficulties of defect identification can be seen in many manufacturing areas; solutions and the newest method applications were presented in [3] with intelligent imaging and analysis techniques applied in various research fields. The authors in [1] reviewed DL network application publications on pavement crack detection since its first appearance in 2016. The importance of the matter can be seen from a recent review paper on pavement defect detection methods [4]. The number of reviewed methods/publications exceeds 100, and half of them are not older than five years. The authors of [4] and [1] showed how important automated visual crack detection for traffic safety is. Unfortunately, the effectiveness of published approaches is often questioned because of the results being demonstrated on publicly unavailable data.

Pavement and concrete have a diversity of surface structures, rubbish, and drawings (lines or figures painted on roads) that hamper the ability to identify true anomalies, namely, cracks. Although a severely damaged surface is fairly easy to spot, defects that start to form are hardly noticeable. There can be found various approaches to pavement defect detection by utilizing different techniques. Older research papers (written five years ago or more) mostly proposed classical image-processing methods to extract road cracks. Researchers in their work use techniques, such as bilevel [5] or histogram-based [6,7] thresholds, to extract decayed pavements’ parts. The authors in [8] used Otsu’s [9] threshold for pavement extraction and Sobel [10] filters along the X and Y directions for crack edge signification and segmentation. The researchers in [6] were interested in detecting extremely noticeable road damage, namely, potholes. Furthermore, a data-driven approach for road pavement evaluation was described by Cord et al. [11]. In [12], the Adaboost machine learning method was used to train image classifiers by pavement patches on a 128 × 128 pixel size for quality inspection. However, they described a drawback of the method when dirt appeared on the image. By looking at more recent research publications, most investigations rely on deep learning methods rather than handcrafted image feature classifiers. Even with small neural network architectures, impressive results compared with other techniques can be achieved [13]. Carr at al. [14] received good results by using a simple U-Net [15] (encoder–decoder) convolutional neural network for defective area segmentation from the CrackForest dataset [16,17]. Another interesting approach was introduced in [18] by utilizing the previously mentioned U-Net and additional outputs to every decoding part layer, and afterwards joining them to improve segmentation. This proposed architectural design significantly boosted the prediction accuracy. Moreover, in the combined method in [19], the authors used preprocessing for a concrete image and engaged in deep neural network-based classification. According to the classification output, the segmentation technique based on a threshold in a 2-D histogram was applied later. Additionally, fully integrated solutions were found for pavement distress segmentation, such as that described in [20]. A vehicle with a camera attached to the top was used to scan the road. The authors proposed an architecture that was not very deep for pavement patch classification. Later on, the same researchers proposed a solution [21] with an extended dataset and improved architectural decisions for road defect detection. They utilized various depth models inspired by ResNet [22] to find the most suitable decision. Research was extended by a more advanced neural network model. The authors were only using 2-D image data, but other approaches can be found for a complete pavement defect detection system (camera on a vehicle) [23] that use 3-D depth data for road structure decay detection. However, steerable matched filter banks are utilized instead of deep learning techniques. Furthermore, fully autonomous systems that utilize deep learning techniques along with computer-vision methods for tunnel concrete quality inspection were described in [24,25]. As can be seen, the interest to cope with infrastructure and pavement defect detection problems is still relevant, aiming to apply DL techniques for real-time automated analysis.

In this work, we continued our investigation on computer vision-based pavement crack segmentation by utilizing a convolutional neural network. This is an extension of our previous work [26] presented at the IDAACS’2019 conference. The mentioned article mainly focused on the classical U-Net encoder–decoder architecture depth (number of convolutional layers) and convolutional filter size dependency on the model prediction precision and computational time. All previous experiments were conducted on the CrackForest dataset. In this paper, we extended our research by utilizing two additional datasets from the similar crack-type area, GAPs384 [18,20,21] and Crack500 [18]. In this work, we present a performance analysis of networks trained on mixed data performance on individual datasets. Small additional adjustments were also made using pretrained weights on the targeted dataset to improve the result. Moreover, we introduced architectural convolutional neural network solutions as an improvement to our previous work. Statistical and visual evaluations were taken into consideration. Neural network implementation and all rendered results can be found in a GitHub repository [27].

The paper is organized as followed. Section 2 explains the neural network and its architectural solution, and methods utilized for research. In Section 3, we describe the CrackForest, Crack500, and GAPs384 datasets, and how we used them for our research. Equipment for the experiments and measurement parameters used for evaluating the neural network performance are outlined in Section 4. Results are given in Section 5. Conclusions and discussion are written in Section 6.

## 2. Deep Neural Network Model

**Figure 1:**
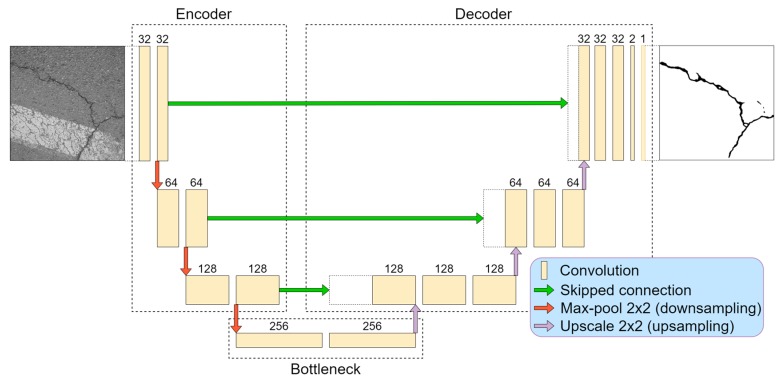
U-Net convolutional neural network structure.

For the baseline in this research, we chose the U-Net [15] deep neural network as an autoencoder function to detect pixel-level cracks in images. The architecture consisted of two main parts, contractive (encoder) and expansive (decoder). The model is shown in Figure 1. In addition to the original structure described in [15], we added padding to the convolutional layers to maintain the output dimension equal to the given input image.

**Figure 2:**
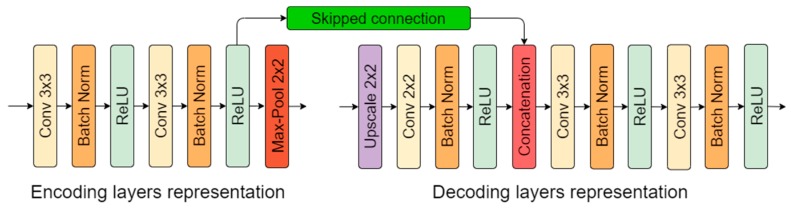
Encoding- and decoding-part layer representation.

From our previous research [26], we took the best-performing solution for crack detection. It was observed that a deeper convolutional neural network tends to learn features more accurately than smaller architectures. A four-layer (eight convolutional layers in the encoder) neural network outperformed two- and three-layer solutions, although close results in smaller models were achieved by using big feature kernels, such as 7 × 7 or 9 × 9. However, big-sized kernel utilization and a small stride significantly slow down the data-processing time. In respect of this, a four-layer neural network was chosen (Figure 1) for this study. All convolutional operations in the encoder part were performed by 3 × 3 kernels with a one-pixel stride. After every two convolutional operations (until the “bottleneck”), dimensions were reduced twice by a 2 × 2 max-pooling operation, and the number of features was therefore doubled. The most contracted part is the “bottleneck” that represents a latent space and has the highest number of convolutional kernels. Further, the decoder or reconstruction part starts (Figure 1). With every layer, the tensor width and height dimension were upscaled twice. Afterwards, a 2 × 2 convolutional operation was performed to adjust and interpret the upscaled data details with the learned parameters. Then, the partly decoded data were concatenated with data from the opposite side (encoder) that transferred higher-level features from the encoder side (see Figure 1 and Figure 2). In all convolutional layers, the rectified linear unit (ReLU) [28] was used as an activation function, and the neural network output (1 × 1 convolution) had sigmoid activation that output the probability of “how likely it is for a pixel to be a defect” in ranges from 0.0 to 1.0. This corresponds to the range from 0 to 255 in 8-bit grayscale. A higher pixel value meant greater confidence that the pixel belonged to a pavement crack.

Additional to previous research [26], batch normalization [29] was added between the convolutional layer and its activation function (except for the last output layer with sigmoid) to increase the neural network stability. When the mean and variance are calculated using batch normalization for a small minibatch compared to the whole dataset (in this case, 4), it gives noise related to the individual iteration. For this mentioned reason, dropout was removed. Weight decay (L2 normalization) was taken out because batch normalization eliminates its effect [30]. The network-encoding and -decoding-layer representation is shown in Figure 2.

**Figure 3:**
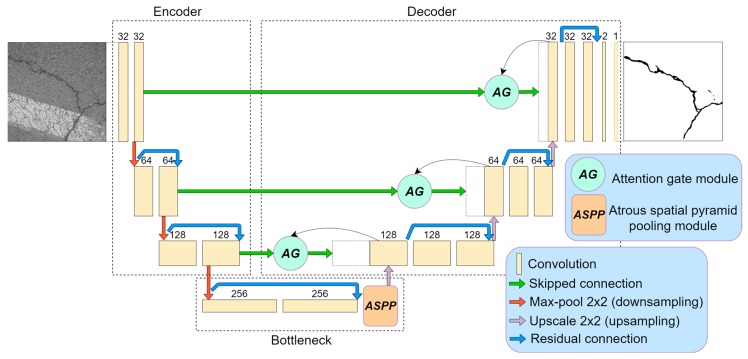
U-Net neural network with few modifications: residual connection, atrous spatial pyramid pooling (ASPP), and attention gate (AG) modules.

Additionally to the classical U-Net architecture, we applied a few architectural improvements to increase the neural performance. The architecture induced with the residual connections, atrous-spatial pyramid pooling (ASPP), and attention gate (AG) modules is shown in Figure 3. Every modification idea is described briefly in the following subchapters. We conducted experiments with several models: U-Net, U-Net with residual connection, U-Net with residual connection and ASPP module (two types), and U-Net with residual connection, ASPP (2 types), and AG modules. The main aspects of this research were computation and prediction–performance difference investigations.

### 2.1. Residual Blocks

**Figure 4:**
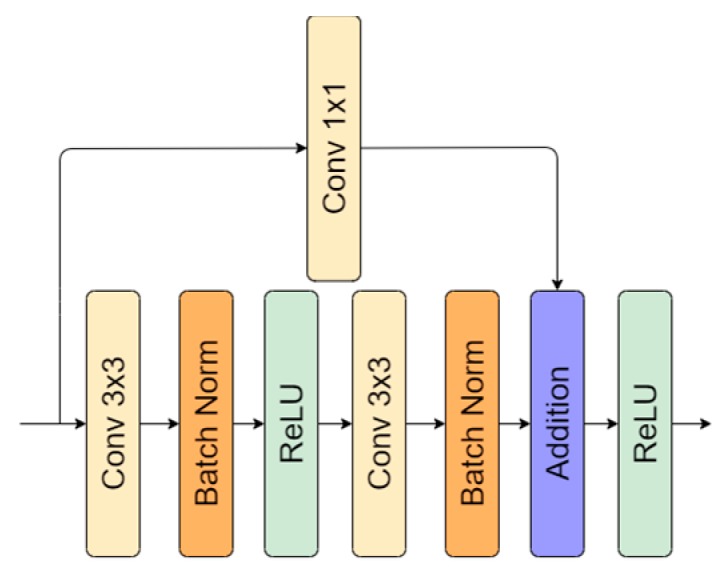
Residual connection representation: 1 × 1 convolutional operation used to adjust number of features.

Architecture induced with residual connection has been used by multiple researchers [31,32,33,34]. It was proven that residual connection helps to fight the vanishing gradient problem, accuracy degradation [35], and improves neural network performance [31,32,34]. Skipped operations also allow undisturbed dataflow through the whole network (Figure 3). In the implementation of the residual connection, we also added 1 × 1 convolution to adjust the number of features because, in every encoding (downscale) or decoding (upscale), the number changes twice. A residual connection with a double convolutional operation is shown in Figure 4. For the present research, we utilized the original implementation of the residual block proposed in [35].

### 2.2. Atrous (Dilated) Convolution Blocks

**Figure 5:**
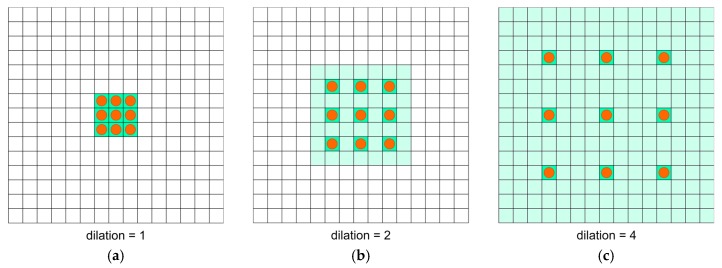
Convolutional 3 × 3 operation with different dilation rates: (**a**)—1, (**b**)—2, (**c**)—4. Orange circles, filter kernel points; green area, contextual image information that might be taken into consideration.

Sequences of dilated convolutions were introduced in [36] as a more capable method to extract semantic information in object segmentation problems. An operation with different dilation rates can take into consideration the multiscale context by utilizing a sequence of convolutions. An example of performing convolutions with different dilation factors is shown in Figure 5. 

**Figure 6:**
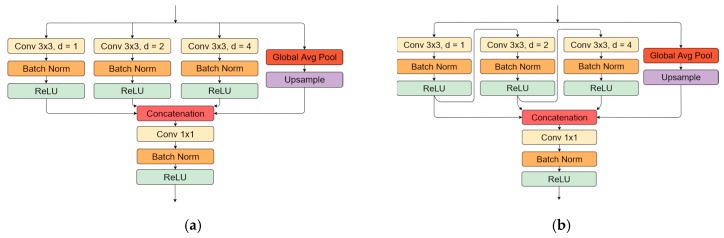
Atrous spatial pooling block representation: (**a**) ASSP block of convolutions with 1, 2, and 4 dilation rates and global pooling in parallel; (**b**) ASPP block with convolutions with 1, 2, and 4 dilation rates connected as suggested in [45] and global pooling in parallel.

Further studies [37,38] proposed an approach of conducting these operations in parallel. Moreover, in [38], global feature tensor pooling in parallel with a convolutional operation was added to capture global context information with each tensor feature layer, as proposed in ParseNet [39]. The described block (ASPP) is used in state-of-the-art object-detection solutions, such as the DeepLabV3 [38] neural network. Models that were induced with this method showed a performance improvement in satellite images [40,41,42], medical [43], and general object segmentation tasks [44]. Additionally, to the existing ASSP module structure, the Waterfall connection sequence was introduced in [45], which reused convolutional operations from different parallel convolution, and it outperformed the original (parallel) implementation in object segmentation tasks. Every convolution operation in parallel branches takes the previous convolution result as an input. In this work, the convolution with 1, 2, and 4 dilation rates was used, considering the small pavement crack scale invariance. The picked values were more likely to be intuitive, and the different choice of dilation factors might yield worst or better results, as was shown in experiments conducted in satellite image segmentation [41]. We tested two types of ASPP blocks:

- As proposed in [38], convolutional operations were performed separately in parallel (Figure 6a); and
- As proposed in [45], input from the previous branch (Figure 6b) was reused for convolutional operations.

### 2.3. Attention Blocks (Attention Gates)

**Figure 7:**
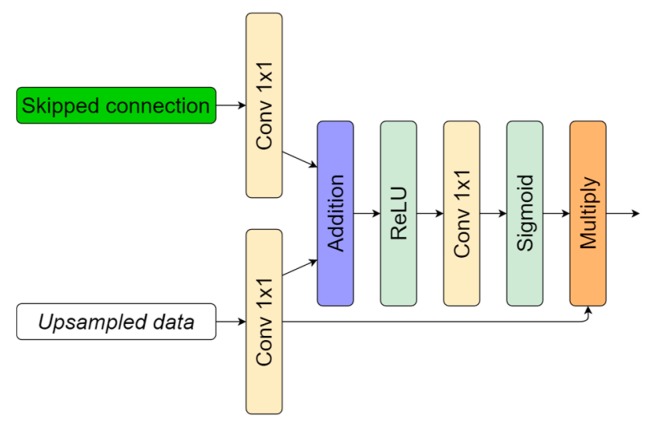
Attention block representation. Upsampled data input taken from the decoder part shown in Figure 2, decoding layer representation before concatenation.

Attention maps were originally proposed in [46] as a technique to improve image classification. Attention modules highlight relevant and suppress misleading information, such as the background. The utilization of such a technique showed an improved U-Net model performance in medical image segmentation tasks [47,48]. In this work, we used attention blocks in the same manner as originally described in [47]. Blocks were implemented in the decoder part before the skipped connection and upsampled-data concatenation. Attention blocks usually amplify relevant information from the previous decoding layer in image reconstruction (decoder part, skipped connection as in Figure 3) and reduce weights on background features. Implementation is shown in Figure 7. As currently drafted, the output of the attention gate is concatenated with upsampled data from the previous layer.

## 3. Data

### 3.1. CrackForest

The CrackForest [2,3] dataset consists of 118 labeled color images taken with an iPhone 5 camera containing noise: Oil stains, road marking, shoe contours, and shadows. Images are 480 × 320 8 bit RGB. Every image has its ground-truth image with its pixel labeling.

**Figure 8:**
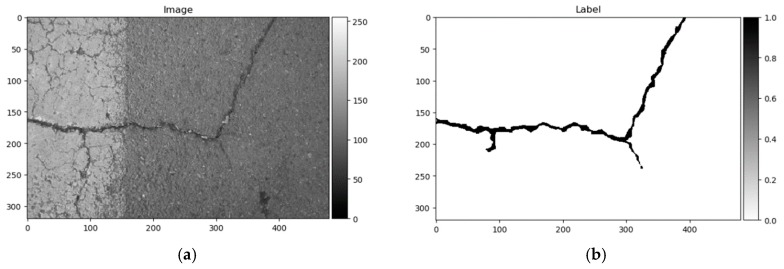
CrackForest data sample. (**a**) Image; (**b**) ground truth.

Label mark 1 corresponds to a good surface; 2, crack; 3, surface enclosed by cracks or area is surrounded by cracks; and 4, narrow, hard-to-see cracks. All 118 images had marks 1 and 2, only 22 images contained pixels with labels 3, and label 4 appeared only in 5 images. The image named 042.jpg mismatched with its corresponding mask image. Label 3 marks were debatable in this dataset, given that they were not equally marked in images, and some elements are differently marked from image to image. Therefore, only the two first classes were used in this research. All 117 images were randomly divided into training and testing sets at 70–30%, respectively—82 images for training, and 35 for testing and converted to greyscale. An example of the data sample can be seen in Figure 8.

Multiple researchers from all over the world used this dataset to practice crack detection, and, according to the researchgate.net portal, the citations of the CrackForest database [16] exceed 108 publications. The variety of applied methods goes from simple rule-based methods [49] to moderate image processing by edge detection [50] or superpixel [51] techniques and histogram features [52] to advanced deep learning-based methods for crack detection [53]. Method evaluation differs from author to author by the used evaluation metrics and strategies, depending on article goals. Some authors proposed to use the tolerance distance from 2 to 5 pixels to overcome data-labeling inaccuracy [14]. The best published results are summarized in Table 1.

### 3.2. Crack500

**Figure 9:**
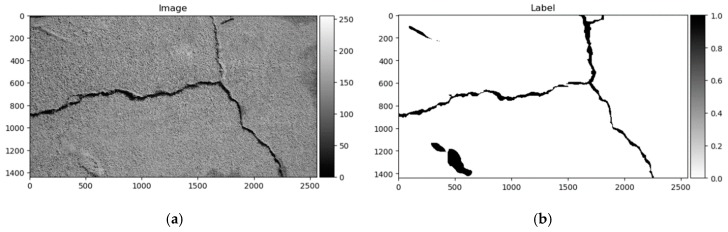
Crack500 data sample. (**a)** Image; (**b**) ground truth.

The Crack500 dataset was introduced in [18], and it contains images taken using cell phones around the main campus of Temple University. It consists of pixelwise annotated pictures around 2000 × 1500 pixels (varying sizes). It has 250 training, 200 testing, and 50 validation samples. As per the authors in [18], it is the biggest pixelwise annotated road pavement defect dataset. Data samples can be seen in Figure 9.

### 3.3. GAPs384

**Figure 10:**
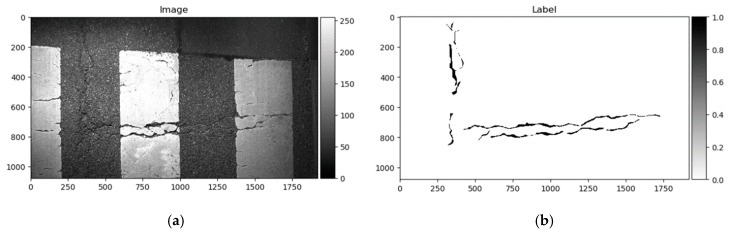
German Asphalt Pavement Distress (GAPs) data sample: (**a**) Image; (**b**) ground truth.

GAPs384 [18] is a derivation of the German Asphalt Pavement Distress (GAPs) dataset proposed in [20,21]. The original dataset is annotated with bounding boxes, while the modified variant (GAPs384) is labeled pixelwise. GAPs384 is part of the GAPs dataset. It provides HD images (1920 × 1080) with a per-pixel resolution of 1.2 × 1.2 mm. GAPs consists of 353 training and 27 testing samples. Pictures were captured in summer 2015 under dry and warm conditions with a specialized mobile mapping system, S.T.I.E.R of Lehman + Partner GmbH. The imaging system consists of two photogrammetrically calibrated monochrome cameras (1920 × 1080 resolution each), while both cameras covered a single driving lane. The GAPs384 dataset consists of cracks, potholes, and filled cracks. Quite challenging samples can be found that include sewer lids, sidewalk rock fragments, rubbish, and worn-off road lines. The big challenge in a particular dataset is non-uniform illumination through the picture. An example of the dataset can be seen in Figure 10.

### 3.4. Data Preparation

In this investigation, we took into consideration three different datasets as described above. Every dataset consisted of different-sized pictures. In Crack500, data samples come in different sizes. Moreover, the image size itself is quite big in the Crack500 and GAPs384 datasets, and it is a problem related to the neural network scalability through a limited amount of computer resources (8 GB graphics-card memory in Nvidia 2070 SUPER). Because of these reasons, data samples were cropped into 320 × 320 px patches with 20 px overlap in all datasets. Prepared data were augmented by rotating by 90, 180, and 270°. However, CrackForest in general contained the smallest number of samples compared with the other two. Additional augmentation with flipping and brightness correction in the range of (–15, 15) was introduced to extend the training part of the CrackForest dataset. The size of the Crack500 samples was reduced twice before image cropping to patches due to the extreme size of images compared with the other datasets.

## 4. Experiments and Evaluation

The neural network algorithm was written in Python (v3.7.4) using Keras API [58] with a Tensorflow 2.0 [59] backend. Experiments were made on a computer with Intel i3 9100F CPU and Nvidia RTX 2070 SUPER 8 GB GPU. Model training and testing were done in a Windows 10 environment.

As described in Section 2, we conducted experiments on several architectures:

- U-Net (Baseline);
- U-Net with residual connections (ResU-Net);
- U-Net with residual connections and ASPP module (ResU-Net + ASPP);
- U-Net with residual connections and ASPP module when connected in “Waterfall” order (ResU-Net + ASPP_WF);
- U-Net with residual connections ASPP and AG modules (ResU-Net + ASPP + AG); and
- U-Net with residual connections ASPP (connected in “Waterfall” order) and AG modules (ResU-Net + ASPP_WF + AG).

In every model training, we picked the combined loss function solution consisting of cross-entropy (Equation (1)) and Dice loss (Equations (2) and (3)). The first part, cross-entropy, is quite often used as a loss function that describes the likelihood of two sets. It can be found in popular machine learning frameworks. Cross-entropy loss is the **X** value relation to the **Ẋ** value in the following expression:

(1) $$L_{CE}=-\frac{\sum_{i=1}^{N}x_{i}\cdot \log\left({\dot{x}}_{i}\right)}{N},$$

where $L_{CE}$ is the cross-entropy loss; $x_{i}$ is the *i*th pixel value in the label matrix **X**; ${\dot{x}}_{i}$ is the *i*th pixel value in the neural network prediction matrix **Ẋ**; and *N* is the total number of pixels.

Another target function is Dice [60] loss. Different than cross-entropy, Dice loss evaluates the overlap of two datasets that are measured in the range from 0 to 1. In image segmentation, the Dice score describes the overlap of sets, label, and prediction:

(2) $$D_{score}=\frac{2\cdot \left|X\;\cap \;Ẋ\right|}{\left|X\right|+\left|Ẋ\right|},$$

(3) $$L_{D}=1-D_{score},$$

where $D_{score}$ denotes the Dice score; X is the label matrix; **Ẋ** is the predicted matrix; and $L_{D}$ is the Dice loss.

The final loss function solution used in this work is expressed in the following equation:

(4) $$L=0.5L_{D}+0.5L_{CE},$$

where L is the loss function; $L_{D}$ is the Dice loss; and $L_{CE}$ is the cross-entropy loss.

**Figure 11:**
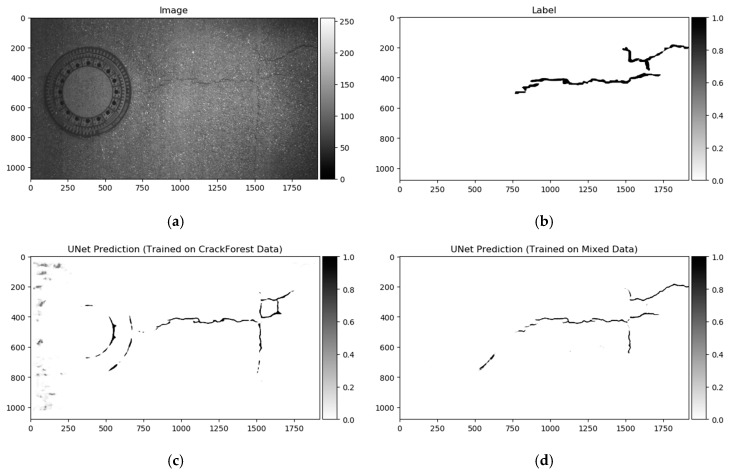
Prediction on the test sample from the GAPs384 dataset. (**a**) Image; (**b**) ground truth; (**c**) full prediction output of the model trained with the CrackForest dataset; (**d**) full prediction output of the model trained with the mixed dataset.

Datasets used in this investigation might not be fully consistent to make for a generalized pavement crack detector for the majority of the cases. We conducted a few experiments on the smallest dataset, CrackForest. The U-Net model was trained for 50 epochs on the CrackForest training set, and tested on GAP384 data (Figure 11c). The model tended to react sensitively to extraneous objects; in this particular case, sewer lids on the road and non-uniform light (image sides in Figure 11a). A small amount of data as in CrackForest proposes a limited amount of general information that is covered in other datasets (GAPs384, Crack500). As a result, the trained model fails to generalize the global context and is not able to distinguish “unseen” objects from pavement defects, although it might be enough to fit the model to the same dataset (make it perform well in the same dataset’s test part). In a few studies [41,55,61], models were pretrained with additional data (or only pretrained the encoding part from segmentation models). The advantage of such weight reuse might be twofold: Faster model training (converging) on the new data, and the ability to improve the generalization and overall prediction performance by introducing more various data with correct labels (as is shown in the comparison table of [45] with models induced with other datasets). We took a similar strategy in this investigation. First, all datasets were mixed for initial network weight training. To equalize every datum from every set, the CrackForest set was additionally augmented by brightness correction and flipping, as was described in Section 3. Models were trained on a mixed dataset for 15 epochs with a 0.001 learning rate at the start, and scheduled reduction by half every 5 epochs. In every epoch, 5636 steps/iteration with a minibatch of 4 were made. Data was shuffled on every epoch start. The model’s performance, trained on a mixed dataset, on the testing sample can be seen in Figure 11d. After training with mixed data, every neural network architecture was trained with every dataset individually for 15 additional epochs with a 0.0005 learning rate at the beginning with a reduction by half every 5 epochs. Only values from the neural network output with a higher or equal to 50% confidence rate were taken into consideration. The best performing solution (according to Dice score) from every training were evaluated with accuracy, recall, precision, Dice score (same formula can be expressed as in Equation (2)), and intersection over union (IoU) measures:

(5) $$Accuracy=\frac{TP+TN}{TP+TN+FP+FN},$$

(6) $$Recall=\frac{TP}{TP+FN},$$

(7) $$Precision=\frac{TP}{TP+FP},$$

(8) $$D_{score}=\frac{2*Precision*Recall}{Precision+Recall},$$

(9) $$IoU=\frac{GroundTruth\;\cap \;Prediction}{GroundTruth\;\cup \;Prediction},$$

where TP is the true positive (correct detection of pixels belonging to labeled defect area); TN is the true negative (nondefective background pixels correctly recognized by detector); FP is the false positive (wrongly detected defect pixels); FN is the false negative (defect pixels undetected by detector); GroundTruth is the labeled image pixels. Precision is the proportion of false alarms; Recall is the proportion of undetected defect pixels; and $D_{\mathrm{score}}$ denotes the Dice score or harmonic mean of the precision and recall.

## 5. Results

**Figure 12:**
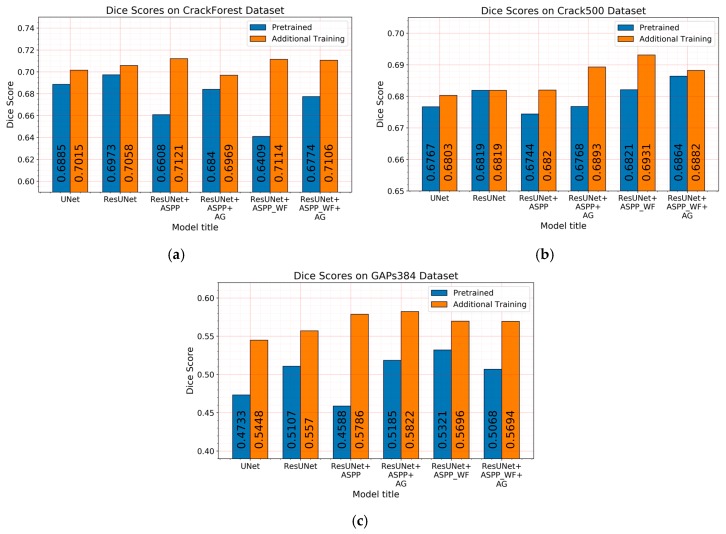
Dice score of every model trained with the mixed dataset (blue bars) and after being trained with a specific dataset (orange bars): (**a**) CrackForest, (**b)** Crack500, **(c)** GAPs384

As in the previous section, at the beginning, models were trained on the mixed dataset, and individually on every separate dataset. The best-performing solutions of models pretrained and additionally trained on the specific dataset Dice score are shown in Figure 12. Each pretrained model weight was the same for every set.

As shown above, the Dice score was improved (in the case of each dataset) with additional training dedicated to the corresponding dataset. The increase in score might be related to the annotation quality or to the experts’ knowledge (that labeled datasets) of problem interpretability. In Figure 8b, Figure 9b, Figure 10b, and Figure 11b show that the details and precision in the sample labels varied. Even in a few annotations from the same dataset (GAPs384, Figure 10b and Figure 11b), the manner of pavement labels might be different. The label in Figure 11b was quite thicker than that in Figure 10b. Training on the mixed dataset in this case possibly ended up fitting the prediction style more or less in favor of one expert (annotation style, such as precision, label line thickness, and other marking properties introduced in specific data sample annotations). Overall, training on the mixed dataset does not highlight a significance in the individual datasets using different architecture neural networks. The increase in Dice was noticeable in most of the cases after short additional training on the individual dataset (Figure 12a–c). Taking U-Net as a baseline for comparison with other models, it is prominent that it has been surpassed by any other architecture. Models induced with a residual connection (ResU-Net) had a slight increase in the Dice score. A more noticeable change can be seen in the GAPs384 dataset that might be related to the data complexity because this dataset introduced more samples with extraneous objects, such as those described in Section 3. Moreover, a big challenge in this particular case is illumination. GAPs384 data require a more powerful model solution for a score increase. Even a larger improvement could be seen by adding an atrous spatial pooling module (ASPP) to the bottleneck of the model. The exact place of addition can be seen in Figure 3. As was described in Section 2, we used two types of links in the ASPP module (Figure 6a,b). Models induced with residual connections and atrous spatial pyramid pooling showed a prediction–performance improvement in the Dice score in all datasets (ResU-Net + ASPP and ResU-Net + ASPP_WF architectures). Models with different types of connections (parallel and Waterfall) in the ASPP part differently favored individual datasets. The biggest increase (from the baseline) could be seen in the GAPs384 set, while the Dice score changed from 0.5448 (U-Net) to 0.5786 (ResU-Net + ASPP). Additionally, we added attention gates (AG) to the ResU-Net + ASPP and ResU-Net + ASPP_WF architectures. However, this enhancement did not always deliver better results. In the CrackForest dataset, the ResU-Net + ASPP + AG and ResU-Net + ASPP_WF + AG neural networks yielded lower results than those of the models without AG modules. On the contrary, the Dice score of the ResU-Net + ASPP + AG architectures surpassed that of the ResU-Net + ASPP in the Crack500 and GAPs384 datasets, and with GAPs384 data, ResU-Net + ASPP + AG achieved the top score.

All measured parameters are given in Table 2, with the highest scores in bold. Accuracy does not represent the prediction performance well because the pavement defects in the image were small, and models performed well by correctly recognizing the background (the biggest part of the image). Intersection over union (IoU) corresponded directly to the Dice score: Models with the highest Dice score delivered the highest IoU. The importance of the false positive (FP) and false negative (FN) costs is described by the recall and precision, respectively. None of these parameters had the top value with U-Net, but in few cases (recall in CrackForest and Crack500 datasets, precision in all datasets), the baseline produced better results than those of some other architecture. Nonetheless, the most important parameter in this investigation was the Dice score, which takes into consideration both recall and precision (as described in Equation (8)).

**Figure 13:**
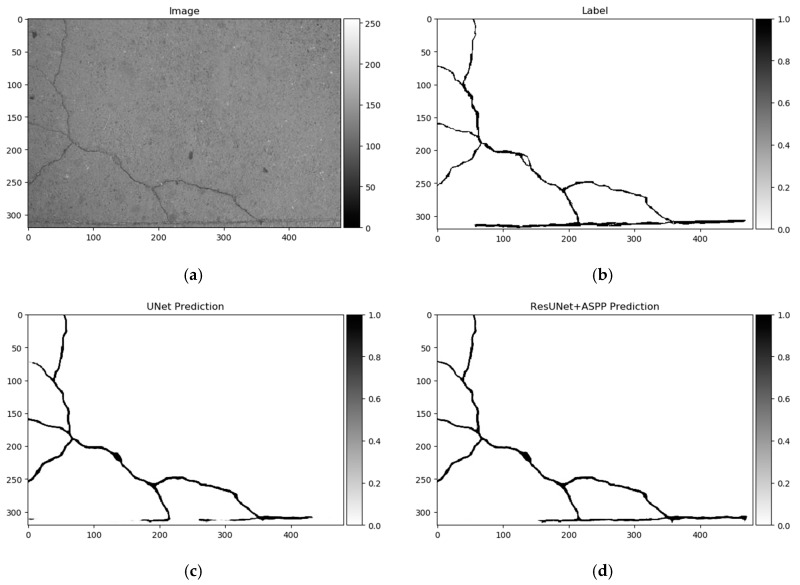
U-Net (baseline) and best-performing solution—ResU-Net + ASPP predictions on CrackForest dataset sample. (**a)** Image, (**b**) label, (**c**) U-Net prediction, (**d**) ResU-Net + ASPP prediction. Segmentation differences significant around image bottom and top left. ResU-Net + ASPP architecture delivered more consistent segmentation.

**Figure 14:**
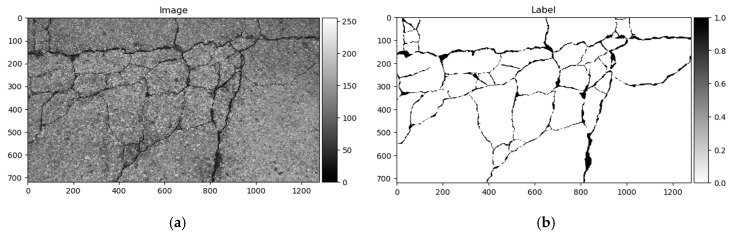

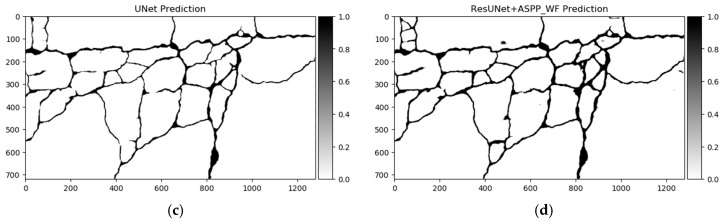
U-Net (baseline) and best-performing solution—ResU-Net + ASPP_WF predictions on Crack500 dataset sample. (**a**) Image, (**b**) label, (**c**) U-Net prediction, (**d**) ResU-Net + ASPP_WF prediction. Quality of continuous defect segmentation noticeable in whole image in predictions (**c**, **d**). ResU-Net + ASPP_WF segmented narrow pavement cracks.

**Figure 15:**
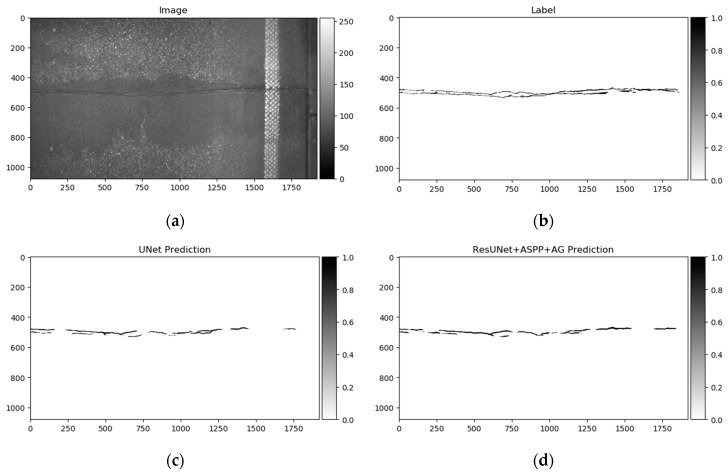
U-Net (baseline) and best performing solution—ResU-Net + ASPP + AG predictions on GAPs384 dataset sample. (**a**) Image, (**b**) label, (**c**) U-Net prediction, (**d**) ResU-Net + ASPP + AG prediction. ResU-Net + ASPP + AG architecture could capture more details compared to U-Net.

Differences in segmentation performance may vary depending on the neural network architectural designs and datasets. The complexity of the analyzed datasets is quite severely altered. Improvements of the Dice scores in different cases are more significant: In GAPs384 between U-Net and ResU-Net + ASPP + AG, and in CrackForest between U-Net and ResU-Net + ASPP. While it can be the main indicator of performance, it is hard to only interpret the segmentation quality from statistical parameters. Representation of the properties, such as the ability to extract a particular feature, for example, narrow defects, can be explained through a visual investigation of the prediction results. As Figure 13, Figure 14 and Figure 15 show, distinctness in pavement defect extraction is noticeable between the baseline (U-Net) and the best-performing solution. The highlight of the better-performing models is the ability to extract hard-to-see indistinctive cracks that the baseline solution fails to do. As it can be seen in Figure 13c, Figure 14c and Figure 15c, U-Net model falls behind in extremely narrow cracks detection compared with models with residual connection and ASSP module (and AG module in Figure 15d) shown in Figure 13d, Figure 14d, Figure 15d. Segmentation continuity is a feature of better detail extraction. In the bottom of Figure 13c and in whole Figure 14c can be noticed that U-Net architecture cannot make continues pavement crack prediction in more complicated cases, while the best-performing solutions shown in Figure 13d and Figure 14d do segmentation with less flaws. More detailed defect extraction is performed by ResU-Net + ASPP + AG model (Figure 15d) compared with the baseline architeture solution (Figure 15c).

**Figure 16:**
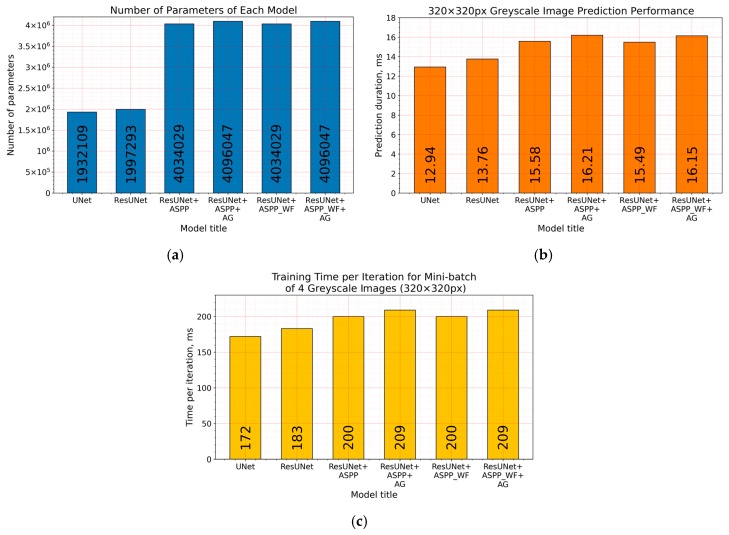
(**a**) Number of parameters, (**b**) prediction computational performance (duration in milliseconds) on a grayscale 320 × 320 px size image, and (**c**) training time per iteration (duration in milliseconds) for the minibatch of four grayscale 320 × 320 px images of each architecture. Performance evaluation and training made with Nvidia 2070S GPU.

While inducing neural networks with additional modules, such as residual connection, ASPP, or AG, we increased the computational complexity (Figure 16a). Extensions enlarged architectures by raising the number of their parameters and by affecting the required time to make the prediction (Figure 16b), taking a bit longer to train the model (Figure 16c). Additional residual connections in U-Net did not make a significant difference, although an increase in the number of parameters was made more than twice by introducing the ASPP module. By adding it in the latent space (bottleneck, Figure 3), we also increased the number of parameters: 256 of 3 × 3 feature kernels in three parallel convolutional operations (Figure 6) for an eightfold downscaled input dimension. However, the number of parameters is not proportional to the computational performance (Figure 16b), and bigger solutions (induced with residual connection and ASPP) took only from 2.55 to 3.27 milliseconds longer to predict in the ResU-Net + ASPP_WF and ResU-Net + ASPP + AG configurations, respectively, compared with U-Net on a 320 × 320 px grayscale-image patch. Inducing models with attention gates did not affect the number of parameters significantly either. Figure 7 shows that it consisted of lightweight 1 × 1 convolutions that did not produce a large computational overhead for the model.

**Figure 17:**
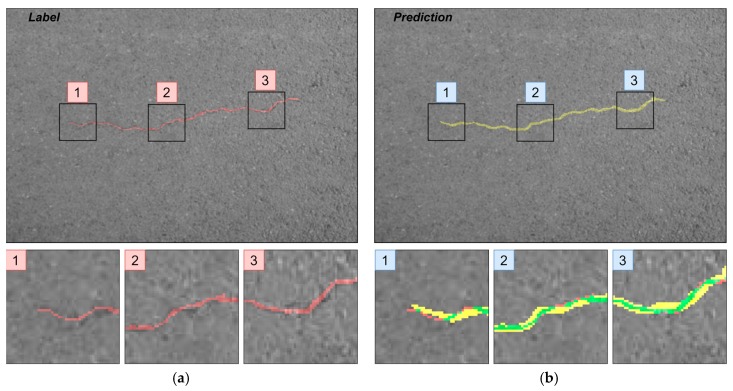
(**a**) Label and (**b**) prediction of U-Net rendered on images from CrackForest and zoomed regions. Green, overlap of label and prediction; red, prediction pixels; yellow, prediction pixels.

Furthermore, the authors in [14] noted that a certain pixel tolerance can be introduced to cope with annotation inaccuracy. In pavement defect labeling, it is hard to define crack boundaries in a complex pattern. As can be seen in Figure 11a,b, Figure 13a,b, Figure 14a,b, and Figure 15a,b, variations in problem interpretation may seem different in different datasets, and crack label thickness can be subjective (Figure 17). This can cause severe deterioration in statistical performance evaluation, especially considering that a crack itself can be narrow, and its area, compared to the background, is small. We introduced a two- and five-pixel tolerance to the statistical evaluation of the best-performing architecture in each dataset; the results are given in Table 3.

A small allowed error of two pixels significantly boosted the segmentation performance, helping to ignore the slight imprecision appearing on the edge of the label (Figure 17). Increasing the tolerance to up to 5 pixels did not make as big an improvement as that of two pixels, although it might depend on the image resolution and detail complexity. In the dataset, when containing higher-resolution images (Crack500 or GAPs384), the Dice score rise is higher. Comparing our results on CrackForest with scores proposed by another (Table 1), our used solution was in first place with the zero- and five-pixel tolerance, and second with the two-pixel tolerance. It is hard to accurately compare results since the CrackForest dataset is not divided into training and testing parts, and forming them randomly can lead to a specific sample correlation favoring the proposed solution.

## 6. Discussion

In this paper, we extended and improved our previous work [26] on pixelwise pavement crack detection by using a convolutional neural network. An investigation of road crack segmentation was scaled up by introducing additional datasets, Crack500 and GAPs384. We also demonstrated architectural improvements to the baseline model, U-Net, which boosted the prediction performance. Network structure enhancements with residual connections, atrous spatial pyramid pooling (ASPP), and attention gates (AG) were experimentally trialed on three different and one mixed datasets. In every dataset case model, the configuration with residual connections and ASPP module outperformed U-Net and ResU-Net. Moreover, the Waterfall connection type in the ASPP module did not favor every dataset. The top result with a particular Waterfall ASPP decision was received in the Crack500 data. The model with the AG module only delivered the highest Dice score in the GAPs384 dataset. This architecture (ResU-Net + ASPP + AG) showed the biggest improvement compared to the baseline (U-Net), with a Dice score improvement from 0.5448 to 0.5822, and with a prediction time on a 320 × 320 greyscale image of 12.94 and 16.21 milliseconds, respectively, using Nvidia 2070S GPU. The introduced pixel tolerance significantly boosted the statistics, up to 0.8219 with two pixels and 0.8966 Dice score, with five pixels of allowed error. Visual segmentation inspection revealed that models induced with residual connections and ASPP modules (and AG modules in few cases) tended to capture more complicated details in pavement patterns, and make segmented cracks more continuous.

Neural network training on a mixed dataset and testing on separate datasets did not deliver consistent results with different architectures. Short additional training on the targeted dataset using pretrained (on mixed data) weights gave a better Dice score. Considering that all three datasets were annotated by different experts, the model could tend to fit one or another problem interpretation presented in the labels that might not favor all datasets. From the described data, the annotation style and details varied. Training only on a limited number of samples (as was described by using the model trained with CrackForest on GAPs384) might not be good at generalization.

In a future work, we are considering revising annotations and introducing even more different data for the problem. As collecting and labeling data samples is time demanding and requires precision, synthetic data might also be introduced in the model learning process. While traditional image-processing methods, such as rotation, brightness correction, and noise addition, can be limited in complicated cases, techniques, such as generative adversarial networks (GANs) or variational autoencoders (VAEs), can be engaged to deal with the particular problem. This showed promising results in recent studies [62,63], and it might be a possible solution for the analyzed task.

## Figures and Tables

**Table 1:** Best results on CrackForest dataset.

Authors	Tolerance in Pixels	Precision	Recall	Dice
Wu et al. [54]	0	0.4330	0.7623	0.4809
Liu et al. [53]	2	0.9748	0.9639	0.9693
Lau et al. [55]	2	0.9702	0.9432	0.9555
Fan et al. [56]	2	0.9119	0.9481	0.9244
Escalona et al. [57]	5	0.9731	0.9428	0.9575

**Table 2:** Each model’s best weight-prediction results on individual datasets.

**CrackForest**	Accuracy	Recall	Precision	IoU	Dice
U-Net (Baseline)	0.9898	0.7465	0.6803	0.5489	0.7015
ResU-Net	0.9901	0.7391	0.6928	0.5546	0.7058
ResU-Net+ASPP	0.9902	0.7474	0.692	0.5603	0.7121
ResU-Net + ASPP + AG	0.9899	0.7271	0.6906	0.5442	0.6969
ResU-Net + ASPP_WF	0.9900	0.7494	0.6896	0.5595	0.7114
ResU-Net + ASPP_WF + AG	0.9896	0.7695	0.6715	0.5575	0.7106
**Crack500**	Accuracy	Recall	Precision	IoU	Dice
U-Net (Baseline)	0.9845	0.7033	0.6996	0.5282	0.6803
ResU-Net	0.9846	0.7002	0.7083	0.5306	0.6819
ResU-Net + ASPP	0.9848	0.6944	0.7152	0.5311	0.6820
ResU-Net + ASPP + AG	0.9841	0.7386	0.6808	0.5389	0.6893
ResU-Net + ASPP_WF	0.9843	0.7524	0.6789	0.5430	0.6931
ResU-Net + ASPP_WF + AG	0.9832	0.7829	0.6447	0.5373	0.6882
**GAPs384**	Accuracy	Recall	Precision	IoU	Dice
U-Net (Baseline)	0.9953	0.4798	0.7231	0.3925	0.5448
ResU-Net	0.9954	0.4957	0.7134	0.4038	0.557
ResU-Net + ASPP	0.9948	0.5754	0.6285	0.4224	0.5786
ResU-Net + ASPP + AG	0.9951	0.5526	0.6675	0.4264	0.5822
ResU-Net + ASPP_WF	0.9955	0.5459	0.7232	0.4179	0.5696
ResU-Net + ASPP_WF + AG	0.9955	0.5251	0.7143	0.4162	0.5693

**Table 3:** Performance evaluation with the 0, 2-, and 5-pixel tolerance on the baseline (U-Net) and best-performing architectural solutions in every dataset.

**CrackForest**	Tolerance, px	Accuracy	Recall	Precision	IoU	Dice
U-Net (Baseline)	0	0.9898	0.7465	0.6803	0.5489	0.7015
U-Net (Baseline)	2	0.9983	0.9797	0.9194		0.9486
U-Net (Baseline)	5	0.9990	0.9994	0.9411		0.9694
ResU-Net + ASPP	0	0.9900	0.7494	0.6896	0.5595	0.7114
ResU-Net + ASPP	2	0.9986	0.9879	0.9280	-	0.9570
ResU-Net + ASPP	5	0.9991	1.0000	0.9472	-	0.9729
**Crack500**	Tolerance, px	Accuracy	Recall	Precision	IoU	Dice
U-Net (Baseline)	0	0.9845	0.7033	0.6996	0.5282	0.6803
U-Net (Baseline)	2	0.9957	0.9403	0.8759	-	0.9070
U-Net (Baseline)	5	0.9982	0.9949	0.9323	-	0.9626
ResU-Net + ASPP_WF	0	0.9841	0.7386	0.6808	0.5389	0.6893
ResU-Net + ASPP_WF	2	0.9960	0.9309	0.9017	-	0.9161
ResU-Net + ASPP_WF	5	0.9986	0.9932	0.9481	-	0.9702
**GAPs384**	Tolerance, px	Accuracy	Recall	Precision	IoU	Dice
U-Net (Baseline)	0	0.9953	0.4798	0.7231	0.3925	0.5448
U-Net (Baseline)	2	0.9979	0.9742	0.6799	-	0.8009
U-Net (Baseline)	5	0.9986	1.0000	0.7772	-	0.8746
ResU-Net + ASPP + AG	0	0.9951	0.5526	0.6675	0.4264	0.5822
ResU-Net + ASPP + AG	2	0.9981	0.9438	0.7280	-	0.8219
ResU-Net + ASPP + AG	5	0.9988	0.9997	0.8127	-	0.8966
